# A Sketch of a Future Policy

**Published:** 1920-05-29

**Authors:** 


### INSANITY AND JUSTICE.

### A Sketch of a Future Policy.

In the first of the Maudsley lectures delivered by Sir James Crichton Browne in the Barnes Hall, Royal Society of Medicine, on Thursday, May 20, attention was most insistently drawn to the urgent necessity for extensive revision of our present rulings on insanity questions, and even though some of the facts quoted have been repeatedly brought forward in the past, the present lecture should do much to convey to those who stand outside the medical circles primarily concerned some invaluable knowledge of the work to be done.

### A Defence.

In the first place the recent attacks by Prof. Eliot Smith were recalled, and the particularly painful picture of the situation then given was to a great extent redrawn, and, we are glad to note, on less pessimistic lines. The lecturer denied that a true element of fairness can be attributed to a sweeping condemnation, to the effect that the ignorance of asylum medical officers in up-to-date knowledge of psychiatry is deplorable, that research is non-existent, and that in employing a system which does not conduce to cure, Great Britain has shirked problems which other continental powers have long ago attacked. In his well-justified defence Sir James cited the fact that the first asylum research laboratory in Europe was established in England, and that nowadays there are several well-equipped establishments from which, once a more settled condition of the country develops, may be expected some of the finest observational work in the world. If we may ourselves subscribe an opinion, the true situation in these matters appears to be exactly parallel to that in which every branch of science coming into intimate contact with legal procedure finds itself at the present period of reconstruction. In short there are a number of legal fetters to be struck off, the medical staff in some of the larger asylums should be reinforced, and the particular branch of the profession more liberally remunerated. Above all, facilities must be made for the *early* treatment of mental disorder. In the past legal formalities connected with asylum treatment, originally intended though they were for the protection of the subject, have indirectly led to "some increase in insanity or rather an accumulation of lunatics." The natural shrinking from certification has been followed by an inevitable passage of a number of cases from the stage of transitory to permanent infirmity, and to a great extent this is the vital point at which change is needed.

### Some Limitations.

On the limitations of those methods by which medicine may help itself out of this *impasse*, Sir James' description may well be quoted. It was in the hope of avoiding such calamities and of furnishing opportunities for the early treatment of cases of acute and recent mental disorders, while at the same time promoting pathological investigations and education in psychology, that Maudsley supplied the funds for the hospital on Denmark Hill. Hospitals like it will spring up in our large towns, and psychiatric clinics will be established in connection with our general hospitals, where sufferers from mild and larval insanity may receive skilled treatment without incurring the odium of having been in an asylum. But there should be an extension of the out-patient department in public asylums and a perfecting of their clinical apparatus, for it is in them, however successful early hospital treatment may be, that an enormous majority of the insane will still be lodged and treated. The early treatment will be successful in intercepting some part of the stream of the mentally deranged that now flows so copiously to our asylums, but exaggerated notions of the benefits that will accrue from it should not be entertained. The main part of that stream consists of congenital idiots and imbeciles, of general paralytics and chronic epileptics, of confirmed cases of *dementia præcox* or paranoia, and of patients labouring under organic or senile dementia, for whom treatment, early or late, is of little avail.

### Comparative Figures.

Another point of interest was made by the comparison between the relatively complete *physical* examination of recruits made by the Medical Boards and the absence of any corresponding inquiry into their mental state. It is a too well realised fact that an enormous number of men of unsound mind passed into the Army, and had we corresponding records of mental unfitness among our male population there is little doubt that their meaning would be just as striking as the physical deficiencies which have been of late again and again brought to our notice. "From the physical returns of stature alone it is possible to infer, having regard to the correlation between height and mental ability, that there was some general deterioration of mental energy," a statement at which some perhaps may cavil, but nevertheless containing more than a grain of truth.

The great moral, however, which the war has brought home to the country is that the whole problem of mental condition and stability is intimately bound up with the general hygiene and physiological existence among the community. It is true that in order that education in these directions may be accomplished, much study of the growing mind is still to be done, but as the lecturer explained, of all the lessons in neurology taught by the war, there have been none more striking than those showing the tendency to natural recovery of nerves that have been concussed, compressed, lacerated, or even divided. If similar recuperative changes occur in the nerve fibres of the brain, it is readily understandable that they too must be gradual, but that

**Insanity and Justice**—(*continued*).

they can occur there seems little doubt. Therefore the time is now ripe not only to study more deeply such strictly scientific phenomena as nervous repair, but also to co-ordinate the country's legal proceedings and control in these matters so that there shall not be one point, one dividing line, between those treated towards cure and those unfortunates who are relinquished as "insane." Rather must a sympathetic, mouldable scheme be gradually evolved whereby every possible opportunity is developed which may encourage the recuperative power of nerve complexes, both in the individual and the national sense.

